# Myocardial fibrosis and arrhythmogenesis in elite athletes

**DOI:** 10.1002/clc.23226

**Published:** 2019-07-04

**Authors:** Sarah A. Ahmad, Nauman Khalid, Evan Shlofmitz, Lovely Chhabra

**Affiliations:** ^1^ Department of Cardiovascular Surgery Saint Francis Medical Center Monroe Louisiana; ^2^ Section of Interventional Cardiology, MedStar Washington Hospital Center Washington District of Columbia; ^3^ Department of Cardiology Heartland Regional Medical Center Marion Illinois

1

To the Editor,

Cuspidi et al present an excellent meta‐analysis of nine studies involving 403 elite athletes and 297 controls investigating volumetric and strain analysis of left atrial (LA) phasic function.^1^ Salient conclusions of their analysis include that the LA volume is higher and LA reservoir and contractile functions are impaired in elite athletes during active training when compared to the controls. The authors also conclude that the LA remodeling and contractile dysfunction may provide a substrate for the development of atrial and ventricular tachyarrhythmias. We wish to highlight a few additional points.

In addition to the LA remodeling that contributes to arrhythmogenesis, there is substantial evidence that endurance athletes are prone to develop myocardial fibrosis (patchy, interstitial, or dense scar) which can also trigger arrhythmias.^2^, ^3^ The myocardial fibrosis can be focal or diffuse and can develop in the right or left ventricle, septum, papillary muscles, or bundle branches.^2^ Cardiac magnetic resonance imaging using late gadolinium enhancement has detected myocardial fibrosis in endurance athletes.^4^ In addition, these findings have been supported by murine models and small post‐mortem studies on human subjects.^2^ Cardiac fibroblasts are normal cell types occurring in healthy hearts and are responsible for extracellular matrix homeostasis, but upon exposure to injury, mechanical stress, or inflammation, these cells may transform into myofibroblasts and induce cardiac fibrosis.^3^ Sequelae of this pathologic remodeling may include myocardial stiffness (increasing ventricular end‐diastolic pressures), chamber dilation, hypertrophy, and ultimately heart failure.^2^, ^3^

The cardiovascular benefits of exercise are well accepted but the tipping point where the physiologic remodeling becomes pathologic is yet somewhat unclear. The authors are to be commended for adding to our fund of knowledge for this burgeoning field. Characterization of at‐risk elite athletes is important because of the risk of arrhythmias and consequently, sudden cardiac death as these may be preventable. A risk prediction model should be established incorporating LA volumetric and strain data from the present study, myocardial fibrosis from the imaging studies, and other established criteria. Future studies should also aim to provide a risk prediction clinical tool to decrease the arrhythmia related morbidity and mortality in elite athletes.

## CONFLICT OF INTEREST

The authors declare no potential conflict of interests.
